# Ultra-Processed Foods and Type 2 Diabetes Mellitus: What Is the Evidence So Far?

**DOI:** 10.3390/biom15020307

**Published:** 2025-02-19

**Authors:** Natalia G. Vallianou, Angelos Evangelopoulos, Ilektra Tzivaki, Stavroula Daskalopoulou, Andreas Adamou, Georgia Chrysi Michalaki Zafeiri, Irene Karampela, Maria Dalamaga, Dimitris Kounatidis

**Affiliations:** 1 First Department of Internal Medicine, Sismanogleio General Hospital, 15126 Athens, Greece; ilektra.papagianni@gmail.com (I.T.); valiadask@gmail.com (S.D.); adamou_andreas@yahoo.com (A.A.); geosamich99@gmail.com (G.C.M.Z.); 2 Roche Diagnostics Hellas S.A., 15125 Athens, Greece; 3 Second Department of Critical Care, Attikon General University Hospital, National and Kapodistrian University of Athens, 12462 Athens, Greece; eikaras1@gmail.com; 4 Department of Biological Chemistry, Medical School, National and Kapodistrian University of Athens, 11527 Athens, Greece; madalamaga@med.uoa.gr; 5 Diabetes Center, First Department of Propaedeutic Internal Medicine, Laiko General Hospital, Medical School, National and Kapodistrian University of Athens, 11527 Athens, Greece; dimitriskounatidis82@outlook.com

**Keywords:** additives, artificial sweeteners, bisphenol-A, type 2 diabetes mellitus, ultra processed foods

## Abstract

Ultra-processed foods (UPFs) are foods that have undergone extensive industrial processing with the addition of various substances in order to make them more tasty, eye-catching, and easy to consume. UPFs are usually rich in sugars, salt, and saturated fat, whereas they lack essential nutrients. The aim of this review is to elaborate upon the current evidence associating overconsumption of UPFs with the development of type 2 diabetes mellitus (T2DM). We will discuss data interconnecting UPFs and T2DM risk and will further describe specific ingredients that have been suggested to increase this risk. In addition, we will thoroughly explain how additives, such as emulsifiers or sweeteners, or other compounds formed during manufacturing, such as acrylamide and acrolein, and during packaging, such as bisphenol-A, are proposed to be implicated in the pathogenesis of insulin resistance and T2DM.

## 1. Introduction

**Figure 1:**
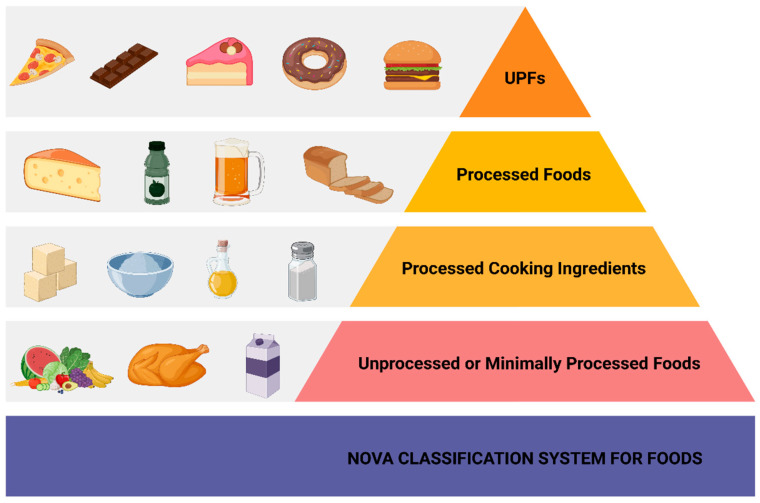
NOVA Classification System: categorizing foods based on the degree of processing. Abbreviations: UPFs: Ultra-processed foods. Created in BioRender. Kounatidis, D. (2025) https://BioRender.com/d50u523. Assessed on 11 January 2025.

Ultra-processed foods (UPFs) comprise foods that have undergone extensive industrial processing, ranging from the addition of sweeteners and emulsifiers to the formation of chemicals during preparation and packaging. UPFs are intended to be more palatable, appealing to consumers, with longer lasting shelf times, and easier to consume [1,2]. According to the NOVA Classification System for Foods, there are four classes of foods: (I) unprocessed or minimally processed foods, which are foods as obtained from plants or animals, (II) unprocessed or minimally processed foods with the addition of salts, sugars, or oils for culinary purposes, or otherwise known as processed culinary ingredients, (III) processed foods consisting of class I or class II foods that undergo further industrial manipulations to make them more tasty and preserved, and (IV) UPFs, which consists of industrial formulations as already aforementioned [1,2]. Figure 1 illustrates the four classes of foods, according to the NOVA Classification System for Foods.

Type 2 diabetes mellitus (T2DM) is a chronic metabolic disorder primarily characterized by insulin resistance (IR) and hyperglycemia. It represents a significant global public health challenge. According to the World Health Organization (WHO), 14% of adults were diagnosed with T2DM in 2022. Additionally, an estimated 59% of individuals over the age of 30 with T2DM were not receiving antidiabetic treatment in the same year [3,4]. Recent evidence suggests that, beyond its well-established association with obesity, the excessive consumption of UPFs may also contribute to an increased risk of T2DM [5,6,7,8,9,10,11].

The aim of this review is to further elucidate upon current data regarding UPF consumption and T2DM risk.

## 2. Human Studies Interconnecting UPFs to T2DM

Snour et al. were the first to explore the association between UPF consumption and the risk of T2DM [12]. They conducted a population-based prospective study with 104,707 individuals over 18 years old from the French NutriNet-Santé cohort, spanning from 2009 to 2019. Dietary intake data were collected through repeated 24-h dietary recalls, which included 3500 food items categorized according to the NOVA Classification for Foods System. Their findings revealed that a higher consumption of UPFs was associated with an increased risk of T2DM (hazard ratio: HR for a 10% increase in UPF consumption = 1.15, 95% CI, 1.06–1.25, *p* = 0.001). This association remained consistent even after adjusting for multiple potential confounders. However, statistical significance was only maintained for women, not men. Notably, women made up 79.2% of the participants, while men comprised 20.8%. The researchers concluded that further large-scale studies in diverse populations and clinical settings are needed to confirm these findings [12].

Levy et al. studied 21,730 individuals from the UK Biobank between 2007 and 2019, aged 40 to 69 years old, and followed them with 24-h recalls by using the NOVA Classification for Foods System, as well [13]. They demonstrated an adjusted HR for each 10% increment in the consumption of UPFs of 1.12, 95% CI, 1.04–1.20. It is noteworthy that there was no gender differentiation regarding statistical power in this study, which included 52.9% women and 47.1% men. Although a statistically significant relationship between increased consumption of UPFs and T2DM risk was observed, public health interventions in the UK, as well as all over the world, should be implemented [13]. In the Seguimiento Universidad de Navarra (SUN) Trial, which followed 20,060 participants for a median of 12 years, 61.5% of whom were women, Llavero-Valero et al. used a Food Frequency Questionnaire (FFQ) that included 136 food items categorized according to the NOVA System. Their findings indicated that a higher intake of UPFs was associated with an increased risk of developing T2DM [14]. Specifically, individuals in the highest tertile of UPF consumption exhibited a significantly greater risk of T2DM compared to those in the lowest tertile (adjusted hazard ratio [aHR]: 1.53; 95% confidence interval [CI]: 1.06–2.22; *p* = 0.024) [14].

In 2022, Duan et al. further explored the heterogeneity of UPFs and their association with an increased risk of T2DM. Their study included 70,421 individuals from the Lifelines cohort, a prospective population-based study in the Netherlands, comprising participants aged 35 to 70 years, of whom 58.6% were women and 41.4% were men. The participants were enrolled between 2006 and 2013, with a median follow-up period of 41 months. The study employed the NOVA classification system to assess dietary intake [15]. Duan et al. reported that a 10-percentage-point increase in UPF consumption was associated with a 25% higher risk of developing T2DM (odds ratio [OR]: 1.25; 95% confidence interval [CI]: 1.16–1.34). Additionally, a principal component analysis identified four distinct UPF subcategory patterns. The findings indicated that a dietary pattern characterized by a high intake of both cold and warm savory snacks was positively associated with an increased risk of T2DM. In contrast, a pattern dominated by sweet snacks and pastries demonstrated an inverse association with T2DM risk (OR: 0.82; 95% CI: 0.76–0.89). Based on these results, the authors concluded that the heterogeneity of UPFs should be carefully considered when evaluating the relationship between UPF consumption and T2DM risk [15].

More recently, Chen et al. conducted a meta-analysis involving three large US cohorts: 71,871 women from the Nurses’ Health Study, 87,918 women from the Nurses’ Health Study II, and 38,847 men from the Health Professionals Follow-Up Study [16]. The researchers used FFQs, the NOVA Classification for Foods System, and the NutriGrade scoring system in their analysis. Their findings showed that a 10% increase in UPF consumption was associated with a 12% higher risk of T2DM. Interestingly, while higher UPF consumption was generally linked to a greater risk of T2DM, some subgroups of UPFs were associated with a lower risk. These results align with those of Duan et al., who also reported heterogeneity in the relationship between UPF consumption and T2DM risk [15,16].

Moreover, the ELSA-Brasil: Estudo Longitudinal de Saúde do Adulto (ELSA-Brasil) trial was a Brazilian longitudinal multicentered study of adult health among 15,105 adults aged 35 to 74 years old in Brazil, followed by an FFQ, using the NOVA Classification for Foods System. In this study, Canhada et al. documented that UPF consumption was related to an increase in the incidence of T2DM. Nevertheless, they also reported a heterogeneity among UPFs, with a more evident relationship between the overconsumption of added sweeteners in natural beverages and T2DM risk [17]. In addition, the European Prospective Investigation into Cancer and Nutrition (EPIC) Study followed 266,666 individuals from seven European countries, comprising 60% women, using FFQs and the NOVA Classification for Foods System. After a follow-up of a median of 11.2 years, the EPIC concluded that UPFs were associated with a higher incidence of cancer, cardiometabolic morbidity, and, especially, T2DM [18]. However, the EPIC Study also pointed out that there was heterogeneity regarding the consumption of UPFs and T2DM risk. In particular, they reported that artificially and sugar-sweetened beverages were correlated with an increased T2DM risk, whereas consumption of UPF cereals and whole grain breads were negatively related to T2DM [18].

In a prospective Korean study involving 7438 individuals aged 40 to 69 years, from the Korean Genome and Epidemiology Study Ansan-Ansung cohort, Cho et al. used a semi-quantitative FFQ comprising 103 items alongside the NOVA Classification System. After a median follow-up of 15 years, they identified an association between higher consumption of UPFs and an increased risk of T2DM. Specifically, they found that the consumption of carbonated beverages (HR: 1.02, 95% CI: 1.00–1.04), ice cream (HR: 1.08, 95% CI: 1.03–1.13), ham/sausages (HR: 1.40, 95% CI: 1.05–1.86), and instant noodles (HR: 1.07, 95% CI: 1.02–1.11) were significantly correlated with increased T2DM risk [19]. Similarly, in 2024, Dicken et al. conducted a prospective cohort analysis as part of the EPIC study, which included 311,892 participants with an average follow-up of 10.9 years. They observed that every 10% increase in daily food intake from UPFs was associated with a 17% higher incidence of T2DM (95% CI: 1.14–1.19) [6]. In contrast, substituting UPFs with foods classified as NOVA classes 1, 2, or 3 resulted in a reduction in T2DM incidence. Dicken et al. also reported heterogeneity among UPF subcategories, noting a lower T2DM risk associated with certain foods, such as bread and breakfast cereals [6].

Very recently, Amirian et al. conducted a study involving 10,047 individuals from the western part of Iran, aged 35 to 65, with a mean follow-up of 7.1 years. Their research, part of the RaNCD (Risk Assessment of Non-Communicable Diseases) study, also found an association between excessive consumption of UPFs and increased T2DM risk. However, after adjusting for multiple confounding factors, the results did not reach statistical significance (*p* = 0.665), prompting the authors to emphasize the need for further large-scale studies to confirm this link [11]. Overall, there is growing evidence supporting an association between higher UPF consumption and T2DM risk [6,11,12,13,14,15,16,17,18,19,20]. Notably, heterogeneity exists among different subcategories of UPFs, with the consumption of sugar-sweetened and artificially sweetened beverages playing a prominent role in T2DM risk. Table 1 summarizes major studies linking UPF consumption to T2DM risk.

**Table 1:** Major studies on the association between UPF consumption and T2DM risk.

Author/Year	Population/Study	Findings	Remarks
Snour et al., 2020 [12]	104,707 participants aged > 18 y.o. (79.2% women) in the French NutriNet-Santé Study, France	A relationship between increased consumption of UPFs and T2DM risk was documented. HR: 1.15 for a 10% increment in the consumption of UPFs.	This study, after adjusting for various confounding factors, showed statistical significance only for women and not for men.
Levy et al., 2021 [13]	21,730 participants aged 40–69 y.o. (52.9% women) from the UK BioBank, UK	aHR: 1.12 for a 10% increment in the consumption of UPFs.	The authors found a statistically significant association between UPF consumption and T2DM risk in men as well as in women.
Llavero-Valero et al., 2021 [14]	20,060 participants, highly educated, aged > 20 y.o. (61.2% women) in the SUN Study, Spain	Higher consumption of UPFs was associated with increased T2DM risk. aHR: 1.53 in the highest tertile of UPFs consumption	This association was observed for both sexes.
Duan et al., 2022 [15]	70,421 participants, aged 35–70 y.o., (58.6% women) in the LifeLines Study, The Netherlands	A diet rich in cold and hot savory snacks was associated with an increased T2DM risk.	Heterogeneity among UPFs was noted in this study. The authors concluded that further studies regarding subcategories of UPFs should be pursued.
Chen et al., 2023 [16]	3 US large cohorts, including 71,871 women, from the Nurses Health Study, 87,918 women from the Nurses Health Study II, and 38,847 men from the Health Professional Follow up Study, with a meta-analysis of these 3 US cohorts	A 10% increment in the consumption of UPFs was associated with a 12% increase in T2DM risk.	The authors documented heterogeneity among UPFs, regarding T2DM risk. Sauces and sweet and artificially sweetened beverages were related to a higher T2DM risk, whereas cereals and dark-whole grain breads were associated with a reduced T2DM risk.
Canhada et al., 2023 [17]	15,105 participants aged 35–74 y.o. in the ELSA-Brasil multicentered study, Brazil	A 150 g/d increment in the consumption of UPFs was associated with a RR:1.05 for T2DM risk.	The authors also documented heterogeneity among UPFs regarding T2DM risk. Sweetened beverages and processed meat were associated with a higher increase in T2DM risk than other UPFs.
Cordova et al., 2023 [18]	266,666 participants (60% women) aged 35–74 y.o. from 7 European countries in the EPIC Study, Europe	Higher consumption of UPFs was related to an increased risk of T2DM.	Heterogeneity was found by the authors. Artificially and sugar-sweetened beverages were the most highly associated UPFs with T2DM risk.
Cho et al., 2024 [19]	7438 participants aged 40–69 y.o. in a Korean study from the Korean Genome and Epidemiology Study Ansan-Ansung Cohort, Korea	During a median follow up of 15 years, an association between the increased consumption of UPFs and T2DM risk was documented.	The authors also demonstrated the existence of heterogeneity among UPFs and the risk of T2DM. A higher consumption of carbonated beverages, ham/sausages, instantly made noodles, and ice-creams was related to an increased risk of T2DM.
Dicken et al., 2024 [6]	311,892 participants aged 35–74 y.o. in the EPIC Study, Europe	A 10% increment in the consumption of UPFs was related to a 17% increase in T2DM risk.	The authors also reported heterogeneity among UPFs and T2DM risk. Breakfast cereals and bread were associated with a reduced risk of T2DM
Amirian et al., 2024 [11]	10,047 participants aged 35–65 y.o. in the RaNCD Study in Iran, Iran	After a mean follow up time of 7.1 years, higher consumption of UPFs was associated with an increased risk of T2DM.	The authors pointed out that further studies are needed to confirm their results, as the statistical power was not significant (*p* = 0.665) after adjusting for multiple confounding factors.
Salame et al., 2024 [21]	104,139 participants > 18 y.o. (79.2% women) in the French NutriNet-Santé Study, France	In this large cohort French study, the authors reported a correlation between food additives emulsifiers and the risk of T2DM.	The authors proposed that legislation regarding food additives should be pursued in order to reduce the burden of T2DM.

Abbreviations: aHR: adjusted hazard ratio; HR: hazard ratio; RR: relative risk; T2DM: type 2 diabetes mellitus; UPF: ultra-processed food; y.o.: years old.

## 3. Proposed Pathogenetic Mechanisms Associating Overconsumption of UPFs with T2DM Risk

Nowadays, there is no established causal link between the consumption of UPFs and the development of T2DM [22]. Despite the fact that no causality has been established so far, there is growing evidence supporting a relationship between overconsumption of UPFs and T2DM risk [6,11,12,13,14,15,16,17,18,19,20,22]. In particular, as already aforementioned, there is heterogeneity among different subcategories of UPFs and T2DM risk. There are several proposed mechanisms associating overconsumption of UPFs with T2DM.

### 3.1. Sugar and Fat Consumption

UPFs are typically high in sugar and fat, both of which are well-known contributors to obesity and IR [5,23]. In a study by Hall et al., 10 participants with stable weight consumed a UPF-rich diet for two weeks, followed by an unprocessed diet for two weeks, while another group of 10 individuals followed the reverse order of diets. The participants were instructed to eat as much or as little as they desired. The results showed that during the UPF-rich diet phase, participants had higher energy intake, with increased consumption of fat (*p* = 0.0004) and carbohydrates (*p* < 0.0001), but no change in protein intake. Notably, they experienced weight gain (*p* = 0.009) during the UPF diet and weight loss (*p* = 0.007) when consuming the unprocessed diet [23]. Based on this controlled intervention at the NIH Clinical Center over four weeks, the authors concluded that reducing UPF consumption could be an effective strategy for preventing and treating obesity [23]. In line with this, Taylor et al. proposed the concept of a Personal Fat Threshold (PFT), which is the level above which excess fat intake cannot be confined to subcutaneous fat storage. They suggested that PFT may be an alternative hypothesis for the development of T2DM irrespective of the Body Mass Index (BMI) [24]. If the PFT is exceeded, accumulation of ectopic fat occurs, mainly in the liver, leading to excess exposure of pancreatic beta cells to fat, over-functioning of beta cells, and ultimately their exhaustion and predisposition to the development of T2DM [24,25]. UPF overconsumption may accelerate the excess of the PFT, thus resulting in T2DM.

### 3.2. Emulsifiers

Apart from their high content in fat and sugars, UPFs have various additives. Amongst them, carrageenan is a common food additive emulsifier, known for its gelling forming and thickening properties, mainly found in ice creams, chocolate milk, and sausages. Carrageenan has very recently been demonstrated to be implicated in IR. In addition, it has been correlated with high serum levels of C-reactive protein (CRP) and interleukin-6 (IL-6) among overweight individuals, thus exhibiting an inflammatory background [26]. In addition, in the same study, carrageenan was found to be involved in increased intestinal permeability, as confirmed by the higher lactulose-to-mannitol ratio [26]. This increased intestinal permeability may lead to the so-called “leaky gut”, with subsequent alterations in the gut microbiota. However, whether changes in the composition and the diversity of gut microbiota occur should be further explored in future studies [26,27].

Until now, emulsifiers, such as carrageenan, agar agar, and polysorbate-80, have been shown in vitro to be associated with decreases in the abundance of *Faecalibacterium* and *Akkermansia genus.* Notably, Naimi et al., by using the MiniBioReactor Arrays, an in vitro model designed to simulate the human gut microbiota, observed significant decreases in the levels of these two microbes. Both *Faecalibacterium* and *Akkermansia* are well-known for their beneficial effects on gut health and their anti-inflammatory properties [28]. As a result, there is increasing interest in the relationship between alterations in the gut microbiota, the overconsumption of UPFs, and the risk of T2DM.

In 2024, Salame et al. documented that food additives, such as total carrageenans (E407-407a), are correlated with the development of T2DM [21]. Specifically, in the French NutriNet-Santé Study, which involved 104,139 participants, it was shown that even though the Acceptable Daily Intake (ADI) of total carrageenans was not exceeded, there was still an association between the consumption of E407-407a and increased T2DM risk. Notably, the ADI of 75 mg/kg for total carrageenans, as assessed by the WHO/Food and Agriculture Organization (FAO) Joint Expert Committee on Food Additives (JECFA), may need to be reconsidered in light of these findings. Additionally, different emulsifiers were found to be linked to cardiovascular disease (CVD) in contrast to T2DM, suggesting distinct metabolic pathways at play in the associations between UPFs and these two conditions [21].

**Figure 2:**
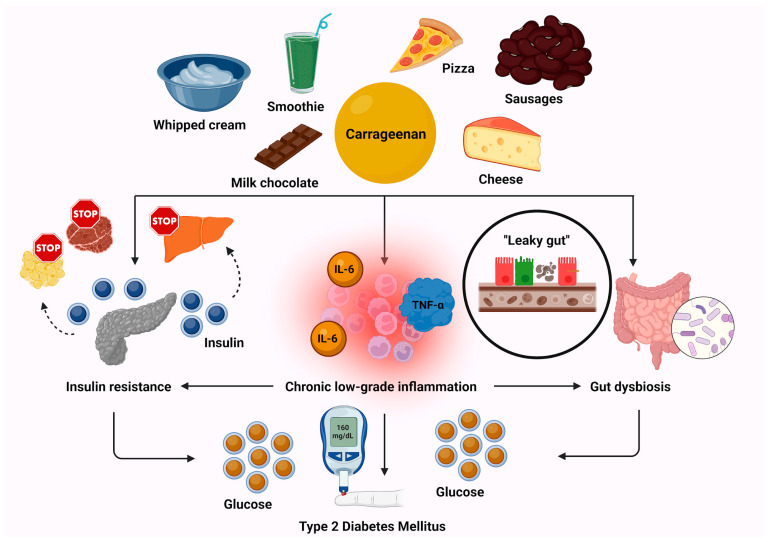
Pathophysiological mechanisms linking carrageenan consumption to T2DM development. Abbreviations: IL-6: intereleukin-6; T2DM: type 2 diabetes mellitus; TNF-α: tumor necrosis factor-alpha. Created in BioRender. Kounatidis, D. (2025) https://BioRender.com/t45g587. Accessed on 7 February 2025.

UPFs and T2DM may be interconnected through insulin resistance and glucose metabolism pathways. Indeed, only recently, carrageenan has been suggested to inhibit the production of proglucagon and glucagon-like peptide-1 (GLP-1) from L-enteroendocrine cells in human cultured L-cells [29]. Further, the administration of carrageenan has already been documented to increase insulin resistance by stimulation of endoplasmic reticulum (ER) stress via selenoprotein P secretion [30]. Apart from total carrageenans, other emulsifiers, like tripotassium phosphate (E340), sodium citrate (E331), guar gum (E412), arabic gum (E414), xanthan gum (E415) and acetyl tartaric acid esters of monoglycerides and diglycerides of fatty acids (E472e), were involved in higher T2DM risk in the landmark study by Salame et al. [21]. Figure 2 illustrates the key pathophysiological mechanisms through which carrageenan consumption contributes to the development of T2DM.

### 3.3. Endocrine-Disrupting Chemicals

It is noteworthy that UPFs may also be contaminated by endocrine-disrupting chemicals (EDCs) during the packaging process, such as phthalates and bisphenols [12,31,32,33]. Phthalates, i.e., esters of pthalic acid, such as di(2-ethylhexyl) phthalate, (PEHP), and diethyhexyl terephthalate (DEHT), are commonly used plasticizers found in UPFs. As plasticizers, they may enter UPFs from plastic containers, especially under prolonged packaging/storage or high temperatures. Phthalates are EDCs, as they have been documented to modulate signaling pathways related to hormone receptors, such as estrogen receptors (ERs), androgen receptors (ARs), and PPARs. Additionally, Yang et al., in 2025, reported on a relationship between increased levels of phthalates and insulin resistance among patients with metabolic dysfunction-associated steatotic liver disease (MASLD) [34]. Regarding bisphenols, bisphenol-A (BPA) is one of the most common EDCs and is the basis of polycarbonate and other plastics. BPA has been found to translocate from packaging into foods due to overheating or an acidic or basic pH environment [31,32,33]. BPA is suggested to be a xenoestrogen due to its estrogen-like potential. Moreover, BPA acts by altering pancreatic beta cell function and by promoting IR in peripheral tissues, thereby increasing T2DM risk [31,32,33]. In particular, BPA has been suggested to alter the function of pancreatic alpha cells that secrete glucagon and pancreatic beta cells, which secrete insulin, thus disrupting the steady-state glucose control [32,33]. Lately, in 2024, BPA has also been called a metabolic disrupting agent (MDA), as it is characterized by 12 key properties, amongst which dysfunction in adipose tissue leading to weight gain, provoking insulin resistance, and cellular stress are maybe the most significant ones [33].

Apart from BPA, acrylamide, an organic compound from the amide family, is also classified as an EDC [35]. In UPFs, acrylamide forms during heating at temperatures above 120 °C as part of the Maillard Reaction (MR), particularly when sugars react with the amino acid asparagine [36,37]. This reaction commonly occurs in foods such as chips, fried potatoes, chocolate, and toasted bread, which are rich in asparagine [36,37]. Acrylamide, as a potential EDC, is thought to have obesogenic properties. It induces adipocyte differentiation and upregulates key metabolic pathways, including the mitogen-activated protein kinase (MAPK) pathway and the AMP-activated protein kinase (AMPK)/acetyl-CoA carboxylase pathway. Additionally, it increases the expression of the adipogenic nuclear transcription factor peroxisome proliferator-activated receptor gamma (PPAR-γ) [38].

Apart from its alleged obesogenic properties, acrylamide has been advocated to be implicated in an increased T2DM risk. More specifically, Hosseini-Esfahani et al. have recently demonstrated that among 6022 individuals from the Tehran Lipid and Glucose Study (TLGS), acrylamide was associated with an increased risk of T2DM in women [39]. In the TLGS, participants were followed for a median of 6.63 years, and acrylamide was documented to be related to an increased T2DM risk in this population. After adjusting for potential confounding factors, the relationship remained statistically significant only for women, in whom there was an increased incidence of T2DM by 13%, and not for men [39].

Furthermore, Wang et al. have also reported that among 3270 adults from the Wuhan–Zhuhai cohort, increased acrylamide exposure, as assessed by specific biomarkers, was associated with increased fasting plasma glucose (FPG), as well as enhancement in oxidative DNA damage and lipid peroxidation [40]. This association was described in the National Health and Nutrition Examination Survey (NHANES) study in the American adult population, which showed that among 1356 adult individuals in the NHANES, between 2003–2004, acrylamide was related to decreased serum levels of insulin [41]. Finally, acrolein, an unsaturated α,β-aldehyde, is another molecule in UPFs suggested to be associated with the homeostasis model assessment-estimated insulin resistance (HOMA-IR) together with fasting insulin levels [42]. In particular, in the NHANES 2005–2006 study, Feroe et al. studied 2027 participants and found a positive relationship with T2DM and IR [42].

Moreover, Wang et al., among 3522 urban adults in a prospective study with repeated measurements during a 3-year follow-up period demonstrated that acrolein was associated with increased T2DM risk by means of lipid peroxidation, oxidative DNA damage, and heme-oxygenase-1 (HO-1) activation [43]. In addition, Wu et al. have proposed that the urinary acrolein–protein-to-creatinine ratio could be utilized as a biomarker of peripheral neuropathy among patients with T2DM. They concluded that further research is anticipated regarding the role of acrolein in diabetic nephropathy [44]. Notably, Jhuo et al. have documented a relationship between acrolein and insulin resistance in vitro and in vivo. More specifically, they found that acrolein induced oxidative stress and mitochondrial dysfunction in muscles and adipose tissue. They pointed out that by scavenging acrolein, we could reduce insulin resistance [45].

**Figure 3:**
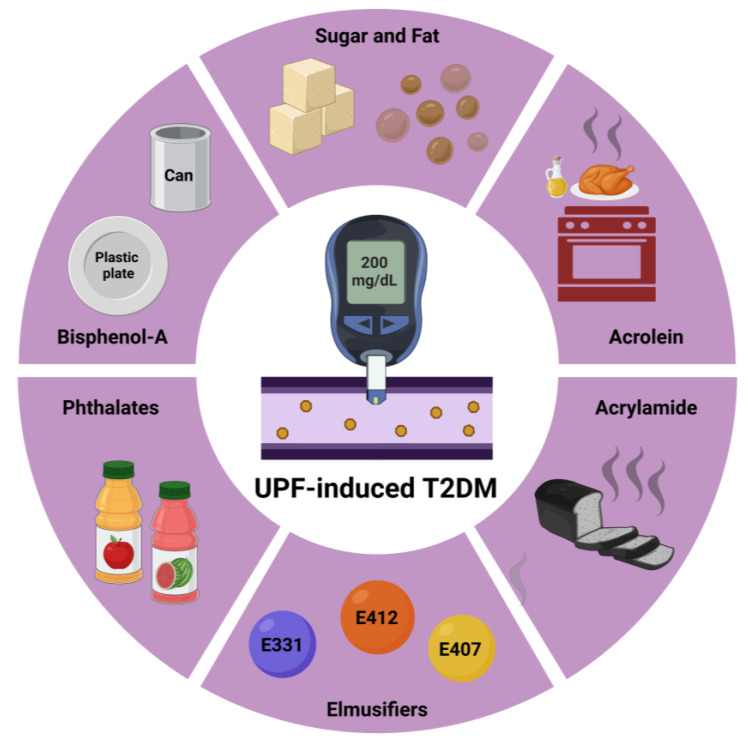
Key components of UPFs associated with the development of T2DM. Abbreviations: E331: sodium citrate; E407: carrageenan; E412: guar gum; T2DM: type 2 diabetes mellitus; UPF: Ultra-processed food. Created in BioRender. Kounatidis, D. (2025) https://BioRender.com/v95c511. Accessed on 18 February 2025.

Overall, although no definite causality has been established until today, the latest study by Salame et al. has shed light on the potential substances in UPFs, which could account for increased T2DM risk. This NutriNet-Santé Study will also examine the plausible alterations in the gut microbiota provoked by specific compounds in UPFs. In the meantime, as UPFs are rich in fat, especially trans-fatty acids and sugar, and rich in additives, the ADI for specific ingredients should not be overlooked and may need to be re-assessed. Figure 3 presents the key components of UPFs associated with the development of T2DM.

## 4. Current Concepts and Future Perspectives

As mentioned earlier, UPFs are usually, but not always, energy-dense and high in saturated and trans fats [45,46]. Additionally, they contain additives that may have harmful effects on cardiometabolic health [46,47]. While UPFs are often hyperpalatable and convenient, their consumption should be limited, as increased intake has been linked to worsening CVD and higher mortality rates [48,49,50,51,52]. There is growing evidence connecting UPFs to an increased risk of T2DM [53,54,55,56,57]. Notably, O’Hearn et al., in their risk assessment model, examined the role of 11 nutritional factors in 184 countries in 2018, compared to 1990, regarding T2DM incidence [57]. Their findings revealed that, in 2018, there were 8.6 million more cases of T2DM than in 1990, primarily due to dietary changes, including decreased whole grain consumption and increased intake of processed meat [57].

Building on the findings regarding cardiometabolic disorders, the American Heart Association (AHA) released dietary recommendations in 2021 aimed at improving cardiovascular health. In these guidelines, the AHA emphasized the reduction of UPFs and their replacement with minimally processed alternatives [58]. In this context, advertising UPFs is highly suggested to continue only under strict legislations and should not be uncontrolled. Nowadays, apart from traditional social media, such as television and magazines, popular social media platforms, which are particularly followed by children and adolescents, are advertising UPFs at alarmingly increasing rates [47,58].

Children’s exposure to these platforms via the internet is on the rise, while children remain much more vulnerable to promotional tips through social platforms when compared to adults [47,58]. Therefore, it is of the utmost importance to protect children from uncontrolled and exaggerated advertising. Many countries have initiated the front of package labeling (FOPL) in foods, but with differential detailing [59,60,61]. The FOPL should be pursued by all countries, and it is advisable to be as detailed as it can [62,63,64,65,66]. Obviously, the FOPL aims at informing mostly adolescents, adults, and the elderly and, to a lesser extent, children. Other measures, such as interventional programs at school or at work, promotion of healthier dietary habits, and strict legislations against one-sided and not pluralistic advertisement, are some paradigms of a multi-faceted approach to this public health issue.

## 5. Life Expectancy in the United States of America: Is There a Connection with UPF Consumption?

Nowadays, the United States of America has the lowest life expectancy among the 12 richest countries worldwide. Despite the fact that the United States of America has started to recover from the COVID-19 pandemic, the life expectancy still remains lower when compared to other economically developed countries. The obesity and overweight epidemic, together with a lack of adequate physical activity, are well-known factors that contribute to this health problem [67]. However, the increased consumption of UPFs is another plausible explanation. Indeed, Juul et al. have reported a significant increase in the consumption of UPFs in the United States during the past two decades. In particular, using data from 24-h recalls among 40,937 adults during 2001 and 2018, they concluded that there was a statistically significant increase in the consumption of UPFs during 2001 and 2018 [68]. Wang et al. also reported that between 33,795 children and adolescents in the United States, an increase in the consumption of UPFs reaching 67% of total energy intake was noted between 1999 and 2018 [69]. In sharp contrast, in other economically large countries with lower rates of non-communicable diseases, the consumption of UPFs accounted for approximately 20% of total energy intake [70,71,72].

Based on the aforementioned data, large-scale well-designed studies are anticipated to further shed light on a potentially causing effect of UPFs on T2DM and other non-communicable disorders. In the meantime, until causality is finally determined or not, it seems prudent that UPF overconsumption should be avoided [73,74,75]. Notably, in 2022, the program “a Healthy Diet for a Healthy Life” has recommended policies on healthier food promotion and a restriction of UPFs, with special emphasis on the widespread availability of fruits and vegetables [76]. Indeed, Silva et al., in their scoping review between 2017 and 2022, have proposed a plant-based diet to promote weight loss and as an effective strategy to prevent becoming overweight/obese and T2DM [77].

## 6. Conclusions

UPF consumption has increased exponentially during the past two decades, especially in the United States of America. There is mounting evidence associating UPF overconsumption with an increased T2DM risk. Although causality is yet to be determined, there is no doubt that strict legislation regarding the use of UPFs should be implemented in various national and multi-ethnic nutritional and medical guidelines. The use of the NOVA Classification System carries endogenous drawbacks, such as heterogeneity, which may limit scientific soundness regarding UPFs research, but is the most widely used nowadays. Therefore, further large-scale and well-designed studies are eagerly anticipated in order to shed light on the relationship between UPF overconsumption and T2DM risk, as most trials have shown heterogeneity in their findings, until today.

## Data Availability

Not applicable.

## Abbreviations

ADI: acceptable daily intake; AHA: American Heart Association; aHR: adjusted hazard ratio; AMPK: AMP-activated protein kinase; ARs: androgen receptors; BPA: bisphenol-A; BMI: Body Mass Index; CI: confidence interval; CRP: C-reactive protein; CVD: cardiovascular disease; E331: Sodium citrate; E340: tripotassium phosphate; E407-407a: carrageenan; E412: guar gum; E414: arabic gum; E415: xanthan gum; E472e: acetyl tartaric acid esters of monoglycerides and diglycerides of fatty acids; EDC: endocrine-disrupting chemical; ELSA-Brasil: Estudo Longitudinal de Saúde do Adulto (Brazilian longitudinal study); EPIC: European Prospective Investigation into Cancer and Nutrition; ER: endoplasmic reticulum; ERs: estrogen receptors; FAO: Food and Agriculture Organization; FFQ: Food Frequency Questionnaire; FOPL: front of package labeling; FPG: fasting plasma glucose; GLP-1: glucagon-like peptide-1; HO-1: heme-oxygenase-1; HOMA-IR: Homeostasis Model Assessment-Estimated Insulin Resistance; HR: hazard ratio; IL-6: interleukin-6; IR: insulin resistance; JECFA: Joint Expert Committee on Food Additives; MAPK: mitogen-activated protein kinase; MASLD: metabolic dysfunction-associated steatotic liver disease; NHANES: National Health and Nutrition Examination Survey; OR: odds ratio; PFT: Personal Fat Threshold; PPAR-γ: peroxisome proliferator-activated receptor gamma; RaNCD: Risk Assessment of Non-Communicable Diseases Study; RR: relative risk; T2DM: type 2 diabetes mellitus; TLGS: Tehran Lipid and Glucose Study; TNF-α: tumor necrosis factor-alpha; UPFs: ultra-processed foods; WHO: World Health Organization; y.o.: years old.
